# Gut bacteria *Akkermansia* is associated with reduced risk of obesity: evidence from the American Gut Project

**DOI:** 10.1186/s12986-020-00516-1

**Published:** 2020-10-22

**Authors:** Qi Zhou, Yanfeng Zhang, Xiaoxia Wang, Ruiyue Yang, Xiaoquan Zhu, Ying Zhang, Chen Chen, Huiping Yuan, Ze Yang, Liang Sun

**Affiliations:** 1 The MOH Key Laboratory of Geriatrics, Beijing Hospital, National Center of Gerontology, Beijing, 100730 People’s Republic of China; 2 University of Chinese Academy of Sciences, Beijing, 100049 People’s Republic of China; 3 Department of Endocrinology, Beijing Hospital, National Center of Gerontology, Beijing, 100730 People’s Republic of China; 4 NHC Key Laboratory of Drug Addiction Medicine, Kunming Medical University, Kunming, 650032 People’s Republic of China

**Keywords:** Gut microbiota, 16S rRNA, Obesity, Body mass index, Probiotics, *Akkermansia*

## Abstract

**Background:**

Gut bacteria *Akkermansia* has been shown an anti-obesity protective effect in previous studies and may be used as promising probiotics. However, the above effect may be confounded by common factors, such as sex, age and diets, which should be verified in a generalized population.

**Methods:**

We used datasets from the American Gut Project to strictly reassess the association and further examined the effect of aging on it. A total of 10,534 participants aged 20 to 99 years from the United States and the United Kingdom were included. The relative abundance of *Akkermansia* was assessed based on 16S rRNA sequencing data. Obesity (body mass index, BMI ≥ 30 kg/m^2^) risks were compared across *Akkermansia* quintiles in logistic models with adjustment for common confounders. Restricted cubic splines were used to examine dose response effects between *Akkermansia*, obesity and age. A sliding-windows-based algorithm was used to investigate the effect of aging on *Akkermansia*-obesity associations.

**Results:**

The median abundance of *Akkermansia* was 0.08% (interquartile range: 0.006–0.93%), and the prevalence of obesity was 11.03%. Nonlinear association was detected between *Akkermansia* and obesity risk (*P* = 0.01). The odds ratios (95% confidence interval) for obesity across the increasing *Akkermansia* quintiles (referencing to the first quintile) were 1.14 (0.94–1.39), 0.94 (0.77–1.15), 0.70 (0.56–0.85) and 0.79 (0.64–0.96) after adjusting for age and sex (*P* for trend < 0.001). This association remained unchanged after further controlling for smoking, alcohol drinking, diet, and country. The odds ratios (95% CI) of *Akkermansia* were 0.19 (0.03–0.62) and 0.77 (0.64–0.91) before and over 40 years, respectively, indicating that the protective effect of *Akkermansia* against obesity was not stable with aging.

**Conclusion:**

High relative abundance of *Akkermansia* is associated with low risk of obesity and the association declines with aging.

## Introduction

Obesity is associated with or even caused by the dysbiosis of gut microbiota [1]. Some beneficial bacteria, such as gut *Akkermansia,* may play a decisive role in reducing the burden of obesity, via modulation of glucose metabolism and low-grade inflammation [2, 3]. Recently, gut bacteria *Akkermansia* has become a very promising anti-obesity probiotic candidate. The genus and its main species *Akkermansia muciniphila* inversely correlated with metabolic syndrome [4, 5]. Treatment with live *A. muciniphila* (dose ~ 10^8^–10^10^ per day) was shown to reduce the risk of obesity and improve insulin resistance, glucose intolerance and steatosis in both mice and humans [6–8]. These findings raise the possibility of new therapeutic strategies for metabolic disorders by targeting the gut microbiota.

However, obesity is an etiologically heterogeneous problem, which is affected by many environmental factors. Such factors can also change the abundance of gut *Akkermansia* and complicate the *Akkermansia*-obesity relationship. For example, *Akkermansia* abundance changes from early childhood to adulthood, varies between geographical locations and differs from health status [4, 5]. On the other hand, aging has also been shown to be strongly correlated with obesity [9], which may easily confound the relationship with gut *Akkermansia*. Indeed, some studies have already revealed inconsistent results such as no significant associations between BMI and obesity [10, 11]. Therefore, before *A. muciniphila* is largely used as a probiotic for treating obesity, three critical questions should be addressed: Firstly, can the potential anti-obesity effects of *A. muciniphila* be generalized to a larger population, especially the elderly? Notably, the effect of *A. muciniphila* on obesity has only been investigated with a small sample size of less than 200 in all prevoius studies. Secondly, how would the common factors such as age, sex, and diet affect obesity and gut microbiota [2, 12–14]? The association changes after adjustment for these confounders remain largely unknown. Thirdly, is there a dose–response effect of *Akkermansia* on obesity or how does the risk of obesity change when *Akkermansia* accumulates in the human gut? To answer these questions, a comprehensive population-scale investigation that takes the common environmental confounders into consideration is needed.

The American Gut Project (AGP) was launched in 2012 to characterizes the diversity of the industrialized human gut microbiome [15]. This project was carried out at an unprecedented scale which included more than 10,000 human participants and yielded over 467 million (48,599 unique) 16S rRNA V4 gene sequencing data. Most importantly, it covers data on microbial compositions in different common factors, and the temporal and spatial stability of gut microbiota, which thereby providing a valuable opportunity to investigate the effect of *Akkermansia* on obesity in a large population under complex environmental conditions.

In this study, we performed a cross-sectional study based on the AGP database of 10,534 subjects. The association between *Akkermansia* and the risk of obesity was estimated with adjusting for age, sex, smoking, alcohol drinking, diet and country. We also investigated the effect of aging on the *Akkermansia*-obesity association.

## Materials and methods

### Study population

This study is a cross-sectional analysis of baseline data from the AGP study, which were initially to explore the relationships between gut microbiota and human health. The health status was self-reported and the microbiota analysis was performed by high-throughput sequencing data of 16S rRNA genes (V4 fragments). The data for the participants from the United Kingdom and the United States were used in this study. Subjects with missing body mass index (BMI); missing sex; BMI > 50 kg/m^2^ or BMI < 10 kg/m^2^; number of qualified sequencing reads < 5000; age < 20 years old were excluded. Finally, a total of 10,534 individuals (5688 females and 4846 males) aged 20 to 99 years were obtained for the present analyses.

### Assessment of obesity

Body mass index (BMI) was calculated as weight in kilograms divided by the square of height in meters. Obesity was identified and classified by BMI according to WHO definitions [16]: normal weight as a reference (BMI < 25 kg/m^2^), overweight (BMI is ≥ 25 but < 30 kg/m^2^), and obesity (BMI ≥ 30 kg/m^2^).

### Assessment of *Akkermansia* abundance and covariates

The relative abundance of *Akkermansia* was obtained by processing the 16S rRNA sequencing reads using Vsearch-2.1.4.2 software with default arguments [17]. Briefly, raw reads were qualified into clean reads with a max error rate of 0.01, clustered into Operational Taxonomic Units (OTUs, similarity threshold 97%), and then contrasted into OTUs tables. Representative OTUs were classified into taxon (sintax_cutoff, 0.8) by using the Silva_16s_v123 database [18]. Relative abundances of *Akkermansia* were assessed by merging the OTUs and taxonomy tables. Samples with < 5000 clean reads were discarded.

Age, sex, smoking frequency, alcohol drinking frequency, diet type, and country (the United States, the United Kingdom.) data were obtained from the baseline questionnaire. Smoking status was grouped into never, occasionally (a few times/month), and regularly (≥ 3–5 times/week). Drinking frequency was classified into never, occasionally (≤ 1–2 times/week), and regularly (≥ 3–5 times/week). The diet types were classified into vegan and nonvegan.

### Statistical analyses

We reported baseline characteristics of this study across *Akkermansia* quintiles. Categorical variables were summarized using absolute numbers and percentages, whereas continuous variables were summarized using the median and interquartile range, or using the mean and standard deviation. Multivariate linear regression or logistic regression were performed for the comparison of common cofounders across *Akkermansia* quintiles.

The correlation between BMI, *Akkermansia* relative abundance and age was tested by Pearson correlation test. Wilcoxon rank-sum test was used to analyze the *Akkermansia* differences between subjects of normal weight, overweight and obesity. Multivariate linear regression or logistic regression was applied for the comparison of common cofounders across the three BMI groups.

Logistic regression models were used to test odds ratios (ORs) and confidence intervals (CIs) of overweight and obesity for each *Akkermansia* quintile compared with the lowest one. Models were first adjusted for age (continuous) and sex and were further adjusted for smoking frequency (ordered category variables), alcohol drinking frequency (ordered category variables), diet type (vegan, nonvegan), and country (United Kingdom., United States.). We performed tests for linear trend by entering the median value of each category of *Akkermansia* relative abundance as a continuous variable in the models. The dose–response relationship was estimated by applying a restricted cubic spline model with 2 knots at the 5th (0.08%) and 50th (3.7%) percentiles of the *Akkermansia* relative abundance.

Stratified analyses were performed according to age (< 65 years, ≥ 65 years), sex, smoking frequency, alcohol drinking frequency, diet type, and country. Likelihood ratio tests were conducted to examine interactions between *Akkermansia* and each of the confounders.

We used a modified SWAN (sliding window analysis) algorithm [19] to identify and quantify nonlinear changes of the *Akkermansia*-obesity association during aging. This algorithm analyzes odds ratios in fully adjusted logistic models within a window of 10 years (e.g., 20–30 years, 30–40 years and so on), while sliding the window in increments of 1 year from young to old. Windows of 5 years and 20 years were also used to compare the robustness of SWAN algorism. Linear regression model was used to test whether the odds ratios were significantly changed with age and group differences of ORs were compared by Mann–Whitney U-tests. For all analyses and plots, we used R × 64 3.6.1 and the related packages. All *P* values were 2-sided.

## Results

### Characteristics of study participants

The characteristics of study participants are presented in Table 1. The average age was approximately 53 ± 15 years, and approximately 54.0% was female. 92.8% of the participants were nonsmokers, 17.5% did not drink alcohol, 6.5% of the sample was vegetarian, and 30.6% of the sample were from the United Kingdom. A total of 26.6% of the participants were overweight, and 11.0% of the participants had obesity. The median relative abundance of *Akkermansia* was 0.08% (interquartile range: 0.006–0.93%), and no *Akkermansia* was detected in 17.6% subjects. Participants who had higher *Akkermansia* relative abundances were more likely to be younger, female, smokeless and nonvegetarians. There was a strong correlation between *Akkermansia* and age: *Akkermansia* abundance was significantly increased with age in an adjusted linear regression model (*P* for trend < 0.001). However, geographic location and alcohol drinking frequency have no significant impact on the abundance of gut *Akkermansia* (both *P* > 0.05). The age, sex, smoking frequency and vegan percentage were significantly different between the three BMI groups (Additional file 1). For instance, the average age of subjects with obesity was 55.5 compared to 50.8 for those with a normal BMI.

**Table 1:** Characteristics of participants according to quintile of *Akkermansia* abundance (n = 10,534)

Characteristics ^a^	Q1	Q2	Q3	Q4	Q5	*P* for trend ^b^
	0–0.003	0.003–0.022	0.022–0.023	0.024–1.40	1.4–49.8	
n	2107	2107	2106	2107	2107	
Age (years)	50.4 ± 15	50.5 ± 14.8	52.5 ± 14.5	53.4 ± 14.7	56.2 ± 14.3	< 0.001
Sex, male, n (%)	988 (46.9)	1079 (51.2)	1024 (48.6)	912 (43.3)	843(40.0)	< 0.001
BMI (kg/m^2^)	24.5 ± 5.2	24.9 ± 4.9	24.7 ± 4.8	24.2 ± 4.5	24.4 ± 4.7	< 0.001
Normal, n (%)	1326 (62.9)	1274 (60.5)	1306(62.0)	1358 (64.5)	1302 (61.8)	
Overweight, n (%)	535 (25.4)	563 (26.7)	553 (26.3)	562 (26.7)	593 (28.1)	
Obesity, n (%)	246 (11.7)	270 (12.8)	247 (11.7)	187 (8.9)	212 (10.1)	
Smoking, n (%) ^c^						0.01
Never	1915(90.9)	1919 (91.1)	1960 (93.1)	1994 (94.6)	1997 (94.8)	
Occasionally	110 (5.2)	120 (5.7)	102 (4.8)	69 (3.3)	66 (3.1)	
Regularly	68 (3.2)	49 (2.3)	37 (1.8)	32 (1.5)	30 (1.4)	
Alcohol drinking, n (%)^d^						0.23
Never	416 (19.7)	390 (18.5)	330 (15.7)	327 (15.5)	385 (18.2)	
Occasionally	1004 (47.7)	1032 (49.0)	1027 (48.8)	1032 (49.0)	981 (46.6)	
Regularly	668 (31.7)	669 (31.8)	740 (35.1)	729 (34.6)	727 (34.5)	
Diet, vegan, n (%) ^e^	134 (6.4)	182 (8.6)	120 (5.7)	138 (6.5)	114 (0.54)	0.02
Country, U.K., n (%)	640 (30.3)	592 (28.1)	657 (31.2)	665 (31.6)	668 (31.7)	0.40

^a^ Data are means ± SD or n (%)

^b^ *P-value* was calculated after adjustment for age, sex, except for itself

^c, d, e^ Data are missing for 66, 77, 124 participants, respectively

^f^ Range of the relative abundances of *Akkermansia* (%)

### *Akkermansia* and risk of obesity

**Fig. 1:**
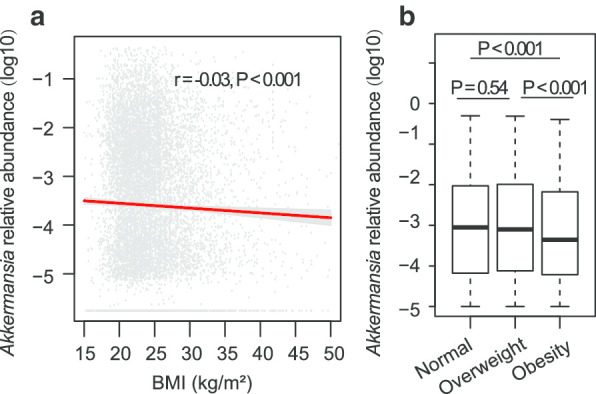
The association between BMI and relative abundance of *Akkermansia*. **a** Pearson correlation between BMI (as a continuous variable) and *Akkermansia* abundance. **b** The Wilcoxon rank-sum test was used to analyze the *Akkermansia* differences between three BMI categories, namely Normal, Overweight, and Obesity. The log_10_-transformed *Akkemancia* relative abundance was used in plots

The BMI and the relative abundance of *Akkermansia* were inversely correlated across the entire study population (*r* = − 0.03, *P* < 0.001) (Fig. 1a), indicating a weak but significant protective effect on gut *Akkermansia* against obesity. Participants with obesity had reduced *Akkermansia* compared to participants with a normal BMI (median relative abundance: 0.088% in normal weight versus 0.043% in obesity, *P* < 0.001) (Fig. 1b). However, the differences between overweight group and normal weight group were not statistically significant (median relative abundance: 0.088% in normal weight versus 0.077% in overweight, *P* = 0.54) (Fig. 1b).

**Fig. 2:**
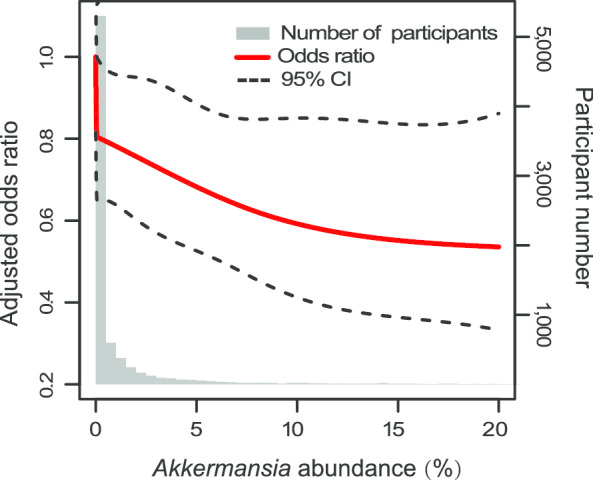
Odds ratio of obesity by relative abundance of *Akkermansia*. Lines represent odds ratios and 95% CI based on restricted cubic splines for relative abundance of *Akkermansia* with knots at the 50th and 90th percentiles. Odds ratios were estimated using a logistic regression model after adjustment for age, sex, diet type, smoking and alcohol drinking and country; *P* for non-linearity = 0.01. Bars represent the numbers of participants according to 100-equally-sized bins of *Akkermansia* abundance

Higher abundance of *Akkermansia* was associated with lower risk of obesity, which was independent of some common confounders (Table 2). After adjusting for age and sex, the ORs (95% CI) for obesity from the lowest to the highest *Akkermansia* relative abundance quintiles were 1.14 (0.94–1.39), 0.94 (0.77–1.15), 0.70 (0.56–0.85) and 0.79 (0.64–0.96) (Model 3). The *Akkermansia*-obesity association was not substantially changed (*P* for trend < 0.001) after further adjusting for smoking frequency, alcohol drinking frequency, diet type, and country (Model 4), suggesting that the protective effect of higher *Akkermansia* abundance against obesity risks is independent of other confounding factors. Furthermore, analysis of dose–response effects revealed a consistent trend toward lower obesity risk for higher *Akkermansia* abundance, and the *Akkermansia-*obesity association was significantly nonlinear (*P* = 0.01) (Fig. 2). However, protective effect was not seen in the overweight group (*P* for trend = 0.13 and 0.15 in Model 1 and Model 2, respectively). The ORs (95% CI) for overweight from the lowest to the highest quantile of *Akkermansia* relative abundance were 1.05 (0.91–1.21), 1.00 (0.87–1.16), 0.98 (0.85–1.13), and 1.04 (0.90–1.21) in Model 1 with adjustment for age and sex. A similar result was found in the fully adjusted Model 2.

**Table 2:** Odds ratio (95% confidence interval) of overweight/obesity according to quintiles of *Akkermansia* relative abundance

	Q1^c^	Q2	Q3	Q4	Q5	P *trend*
	0–0.003	0.003–0.022	0.022–0.023	0.024–1.40	1.4–49.8	
Overweight / Normal (n)	535/1326	563/1274	553/1306	562/1358	593/1302	
Model 1^a^	1	1.05 (0.91–1.21)	1.00 (0.87–1.16)	0.98 (0.85–1.13)	1.04 (0.90–1.21)	0.13
Model 2^b^	1	1.06 (0.91–1.23)	0.99 (0.86–1.16)	0.98 (0.84–1.13)	1.04 (0.89–1.20)	0.15
Obesity / Normal (n)	246/1326	270/1274	247/1306	187/1358	212/1302	
Model 3^a^	1	1.14 (0.94–1.39)	0.94 (0.77–1.15)	0.70 (0.56–0.85)	0.79 (0.64–0.96)	< 0.001
Model 4^b^	1	1.16 (0.97–1.42)	0.96 (0.79–1.17)	0.71 (0.57–0.87)	0.78 (0.63–0.95)	< 0.001

^a^Model 1 and Model 3 were adjusted for age and sex

^b^Model 2 and Model 4 were additionally adjusted for diet type, smoking and alcohol drinking and country

^c^Range of the relative abundances of *Akkermansia* (%)

**Fig. 3:**
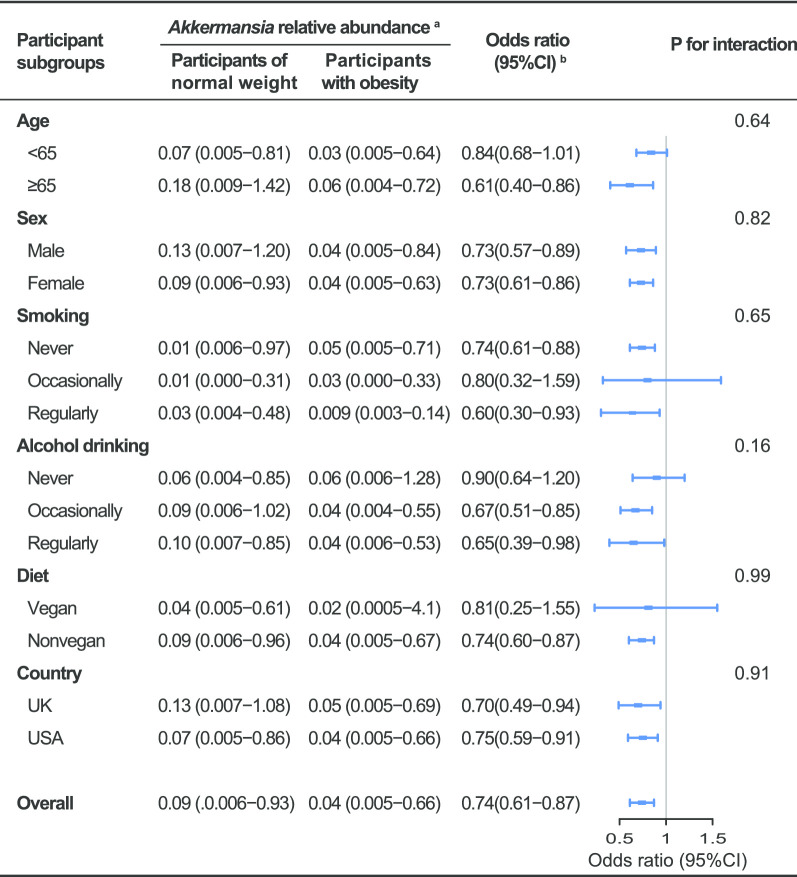
Stratified analyses of the associations between fecal relative abundance (%) of *Akkermansia* and obesity. ^a^Range of the relative abundances of *Akkermansia* was revealed by median (interquartile range). ^b^Adjusted for age, sex, diet type, smoking and alcohol drinking and country, stratifying factors excepted

To confirm the above observed *Akkermansia*-obesity association, stratified analysis was performed (Fig. 3). The association can still be observed when common factors were stratified. However, it was slightly stronger in the elderly, smokers, alcohol drinkers, and non-vegetarians, but no significant interactions were detected with these variables (*P* for interaction > 0.1).

We finally calculated the risk of obesity in a fully adjusted model with *Akkermansia* as a continuous variable. The results showed that 10% higher *Akkermansia* would reduce the risk for obesity approximately 26%, on average (OR: 0.74, CI: 0.61–0.84) (Fig. 3). Risks of overweight for a 10% increase of *Akkermansia* was also estimated. In line with the above results, the association between *Akkermansia* and overweight was not statistically significant (*P* = 0.1).

### Decreased anti-obesity effect of *Akkermansia* with aging

Aging has been known as a strong confounder of the *Akkermansia*-obesity association. In the present study, BMI and *Akkermansia* were both positively correlated with age (BMI: r = 0.13, *P* < 0.001; *Akkermansia*: r = 0.07, *P* < 0.001) (Additional file 2a, b). Besides, a significant nonlinear association was also detected between age and obesity risks (*P* for nonlinearity < 0.001) (Additional file 2c).

**Fig. 4:**
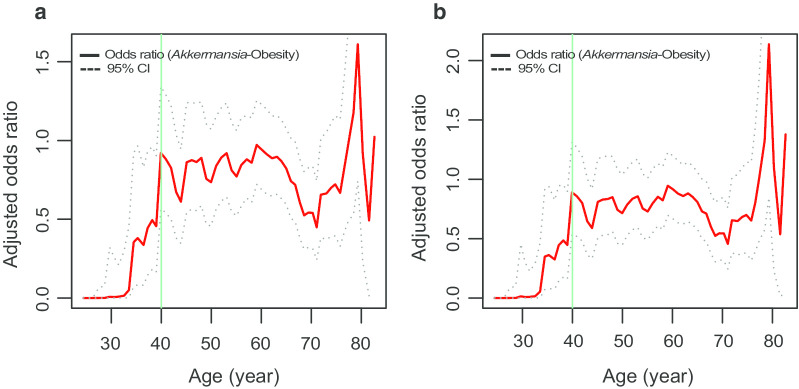
The effect of aging on *Akkermansia-*obesity associations. **a** The changing ORs (between *Akkermansia* and obesity) with age in sliding window analysis. The OR represented obesity risks of elevating of per 10% *Akkermansia* abundance in age- and sex-adjusted (**a**), or in fully adjusted logistic regression models (**b**). Lines represent odds ratios and 95% CI

To further understand the aging effects, we compared the alterations in obesity risks after a 10% increase in abundance of *Akkermansia* across different age groups using SWAN algorithm. The results showed that aging weakened the protective effect of *Akkermansia* against obesity (Fig. 4). The OR value significantly increased with age and reached a plateau at approximately 40 years old in models controlled for age and sex (before 40 years old, *P* < 0.0001; after 40 years old, *P* = 0.709) (Fig. 4a) or models that additionally controlled for the other confounders (before 40 years old, *P* < 0.0001; after 40 years old, *P* = 0.08) (Fig. 4b).

The protective effect of *Akkermansia* before 40 years was significantly smaller than after 40 years (Table 3, Additional file 3): In fully adjusted models (Model 2), the ORs (95% CI) in subjects of < 40 years and ≥ 40 years were 0.19 (0.03–0.62) and 0.77 (0.64–0.91), respectively, and ORs (95% CI) in Model 1 were 0.20 (0.04–0.65) and 0.78 (0.64–0.92), respectively, in the two age groups. To confirm the robustness of the SWAN algorithm, we set the width of the slide window to 5 years or 20 years, and consistent findings were observed between different models (Additional file 4).

**Table 3:** Odds ratio^#^ (95% confidence interval) of obesity before and after 40 years old

	Age groups (year)	
	20–39	40–99
BMI	23.3 ± 4.2	24.9 ± 5.0
Normal, n, (%)	1752 (74.7)	1752 (58.8)
Overweight, n, (%)	445 (19.0)	445 (28.8)
Obesity, n, (%)	149 (6.4)	149 (12.4)
Model 1^a^, OR (95% CI)	0.20 (0.04–0.65)^***^	0.78 (0.64–0.92)
Model 2^b^, OR (95% CI)	0.19 (0.03–0.62)^***^	0..77 (0.64–0.91)

^a^Model 1 was adjusted for sex

^b^Model 2 was additionally adjusted for diet type, smoking and alcohol drinking and country

^#^The Obesity risk of elevating 10% abundance of *Akkermansia*

^***^OR differences between age groups were significant with *P* values of < 0.0001 by Mann–Whitney *U*-test

## Discussion

To our knowledge, this study is the first large population-based study showing that decreased gut *Akkermansia* was associated with an increased risk of obesity, which is independent of age, sex, smoking, alcohol drinking, diet and country. We also found that the association changed with aging.

Most previous studies found that the depletion of *Akkermansia-like* spp. was strongly associated with obesity and metabolic disorders, but some others showed that the level of *A. muciniphila* did not correlate with BMI value [10, 11]. Our large-scale population estimates showed that increased BMI and risk of obesity were correlated with decreased relative abundance of *Akkermansia.* Such discrepancy may come from some confounders, especially when the sample size was small. Many common factors such as age, sex, geography and ethnic origins shaped the gut microbiota [13, 20, 21], and were closely related to obesity. Therefore, it is necessary to assess the independent effect of *Akkermansia* on obesity in large populations. We found that the association between the risk of overweight and *Akkermansia* relative abundance was not statistically significant in all of the models. No relevant results were mentioned in animal or human studies. The “Enterotype” of normal weight subjects is more similar to that of overweight subjects than that of subjects with obesity according to *Akkermansia* distribution.

We adjusted for age and sex in all models because these factors influence obesity prevalence and the gut microbiota [11, 13]. Age shifted the core gut microbiota in both composition and diversity and may confuse relationships between microbiota and diseases. One previous report showed that the aging-associated microbiome masked the microbial signatures of colorectal cancer because of the interactions between cancer-microbiome and aging [22]. The *A. muciniphila* abundance was significantly increased from early life to adult subjects and then reduced in elderly subjects [23]. We found that the protective effect of *Akkermansia* decreased with aging: it kept decreasing from young adults to middle-aged (40 years old). This result indicates that aging may weaken the protective effect which deserves attention in further experiments or clinical trials. The risks of obesity between genders were not statistically different in our study.

We also found a nonlinear association between obesity risk and *Akkermansia* abundance in two models where the relative abundance of *Akkermansia* was treated as continuous or categorical variables, respectively. This result has not been mentioned before. The *Akkermansia* dose–response effects in metabolic dysfunction have been noted in animal models: the live *A. muciniphila* must be used at doses > 4.0 × 10^7^ CFU/day to exert their beneficial effect [24]. Other interventional studies, in humans and mice, used 10^8^–10^10^ CFU/day to improve metabolic diseases [7, 8]. There are no published clinical trials of *A. muciniphila* in humans, which results in a lack of evidence on what dose of the bacteria is beneficial and safe in humans. Administration of *A. muciniphila* at 10^8^ CFU/day has been proved to be safe for humans [7], and there were no adverse events that occurred in people whose relative level of *A. muciniphila* reached 60% [25]. We observed a significant reduction of obesity risk when the relative abundance of *Akkermansia* reached the fourth quintile (0.24%).

One previous study reported that *A. muciniphila* was detected in 75% of European populations [23], while we found ~ 82% of the populations had *Akkermansia*. The frequency of *A. muciniphila* was lower in Southern Chinese (~ 52%) [26] than the European population. However, the *Akkermansia* was similar between people in the United Kingdom (~ 82%) and the United States (~ 83%) in the present study. These results could be due to the differences between the Western and Eastern diets. The median relative abundance of gut *Akkermansia* in our population was 0.08% (interquartile range: 0.006–0.9%), while in the other studies, the relative abundance of *A. muciniphila* was significantly different, accounting for < 0.01–5% [5, 6, 14]. The explanation for the wide range of *Akkermansia* and *Akkermansia-like* bacteria is not known. It may result from different methods for bacterial identification (e.g., in *situ* hybridization, qPCR sequencing, whole-genome shotgun sequencing and 16S rRNA sequencing). The high-throughput sequencing, which detects more unculturable bacteria, may result in a lower relative abundance of *Akkermansia* than the other methods.

As with any observational study, there are several limitations in the present study: (i) Clinical records on some confounders such as cancer, hypertension, and treatment with metformin were not available. For example, metformin administration increased *Akkermansia* abundance in obese mice and type 2 diabetic subjects [27, 28]. (ii) Our findings were based on subjects who consumed the Western diet, which may reduce the generalizability of this result. Although we adjusted the diet type (vegan or nonvegan), validation in subjects consuming Eastern diet is recommended. Besides, due to the nature of self-reported data, the classification of some factors, such as smoking frequency may induce errors into the analysis. (iii) The *Akkermansia*-obesity association (genus level) does not represent the association between *A. muciniphila* and obesity (species level). *A. muciniphila* is the major species in the genus of *Akkermansia*, and both of them exhibit strong associations with obesity. We focused on *Akkermansia,* because the species level may not be accurately annotated by sequencing the 16S rRNA V4 fragment. The genus-level rather than the species-level is frequently preferred in high-throughput 16S rRNA sequencing studies.

Our study has several key strengths. First, the sample size (> 10,000 participants) is larger than any of the previous studies, to our knowledge. Second, the adjustment of several important confounders especially age in models provided a clearer understanding of the *Akkermansia*-obesity association than previous studies. Third, it is the first analysis that systematically reveals the undulating changes in *Akkermansia*-obesity relationships across the lifespan.

## Conclusions

Based on a large dataset from AGP and by accounting for common confounders such as sex, age and diet, we provided evidence that a higher relative abundance of *Akkermansia* may be associated with a low risk of obesity and this association seems to decline with aging.

## Supplementary information

**Additional file 1**. Characteristics of participants among BMI groups.

**Additional file 2**. The correlations of age with *Akkermansia*, BMI and obesity risk.

**Additional file 3**. The differences of ORs before and after 40 years old in age-and sex-adjusted and fully adjusted models.

**Additional file 4**. The effect of aging on *Akkermansia*-obesity associations using SWAN algorithm with 5-years-, 10-years- and 20-years-sliding windows.

## Data Availability

The data of this study were downloaded from EBI under project PRJEB11419.

## Abbreviations

BMI: Body mass index
OTUs: Operational Taxonomic Units
95% CI: 95% Confidence interval
OR: Odds ratio
SD: Standard deviation
SWAN: Sliding window analysis
AGP: American Gut Project
