# Supplementary material for: Gut bacteria Akkermansia is associated with reduced risk of obesity: evidence from the American Gut Project
Source: Nutr Metab (Lond). 2020 Oct 22;17:90. doi: 10.1186/s12986-020-00516-1 (PMC7583218; doi:10.1186/s12986-020-00516-1)
Supplement: Supplementary file 1 — Additional file 1. Characteristics of participants among BMI groups. [file 12986_2020_516_MOESM1_ESM.docx]

**Additional file 1.** Characteristics of participants among BMI groups (n=10,534)

| Characteristics ^a^ | Normal weight | Overweight | Obesity | P for trend^b^ |
| --- | --- | --- | --- | --- |
|  | < 25 kg/m^2^ | 25- 30 kg/m^2^ | ≥30 kg/m^2^ |  |
| n, (%) | 6566(62.3) | 2806(26.6) | 1162(11.0) |  |
| Age (years) | 50.8±15.2 | 55.5±14.1 | 55.5±13.0 | <0.001 |
| Sex, male, n (%) | 2647(40.3) | 1704(60.7) | 495(42.6) | <0.001 |
| Smoking, n (%) ^c^ |  |  |  | 0.003 |
| Never | 6157(93.8) | 2612(93.1) | 1082(93.1) |  |
| Occasionally | 295(4.5) | 128(4.6) | 44(3.8) |  |
| Regularly | 114(1.7) | 66(2.3) | 36(3.1) |  |
| Alcohol drinking, n (%) ^d^ |  |  |  | 0.84 |
| Never | 1236(18.8) | 370(13.2) | 242 (20.8) |  |
| Occasionally | 3029 (48.9) | 1234(44.0) | 633 (54.5) |  |
| Regularly | 2070 (31.5) | 1181(42.1) | 282 (24.3) |  |
| Diet, vegan, n (%) ^e^ | 518 (7.9) | 117 (4.2) | 53 (4.6) | < 0.001 |
| Country, U.K., n (%) | 2043(31.1) | 811(28.9) | 368 (31.7) | 0.61 |

^a^ Data are means ± SD or n (%).

^b^ *P-value* was calculated after adjustment for age, sex, except for itself.

^c, d, e^ Data are missing for 66, 77, 124 participants, respectively.
