# Supplementary material for: Gut bacteria Akkermansia is associated with reduced risk of obesity: evidence from the American Gut Project
Source: Nutr Metab (Lond). 2020 Oct 22;17:90. doi: 10.1186/s12986-020-00516-1 (PMC7583218; doi:10.1186/s12986-020-00516-1)
Supplement: Supplementary file 2 — Additional file 2. The correlations of age with Akkermansia, BMI and obesity risk. [file 12986_2020_516_MOESM2_ESM.docx]

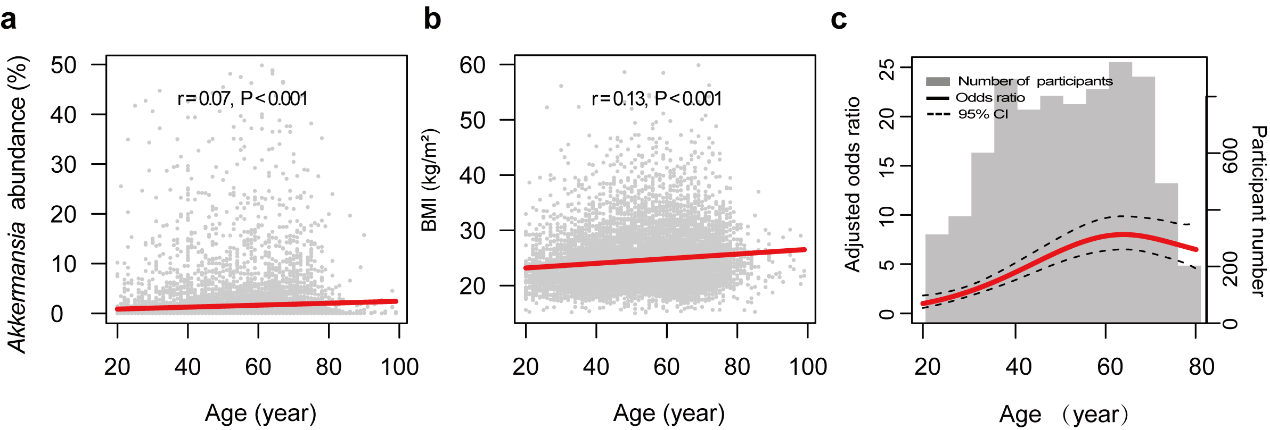


**Additional file 2.** The correlations of age with *Akkermansia* (a), BMI (b) and obesity risk (c). (a, b) Correlation analysis were performed by Pearson correlation test. (c) The non-linear association between age and obesity risk in fully adjusted logistic models with restricted cubic splines (P for non-linearity < 0.001). Lines represent odds ratios and 95% CI. Bars in represent the numbers of participants according to 12-equally-sized bins of age.
