# Supplementary material for: Gut bacteria Akkermansia is associated with reduced risk of obesity: evidence from the American Gut Project
Source: Nutr Metab (Lond). 2020 Oct 22;17:90. doi: 10.1186/s12986-020-00516-1 (PMC7583218; doi:10.1186/s12986-020-00516-1)
Supplement: Supplementary file 3 — Additional file 3. The differences of ORs before and after 40 years old in age-and sex-adjusted and fully adjusted models. [file 12986_2020_516_MOESM3_ESM.docx]

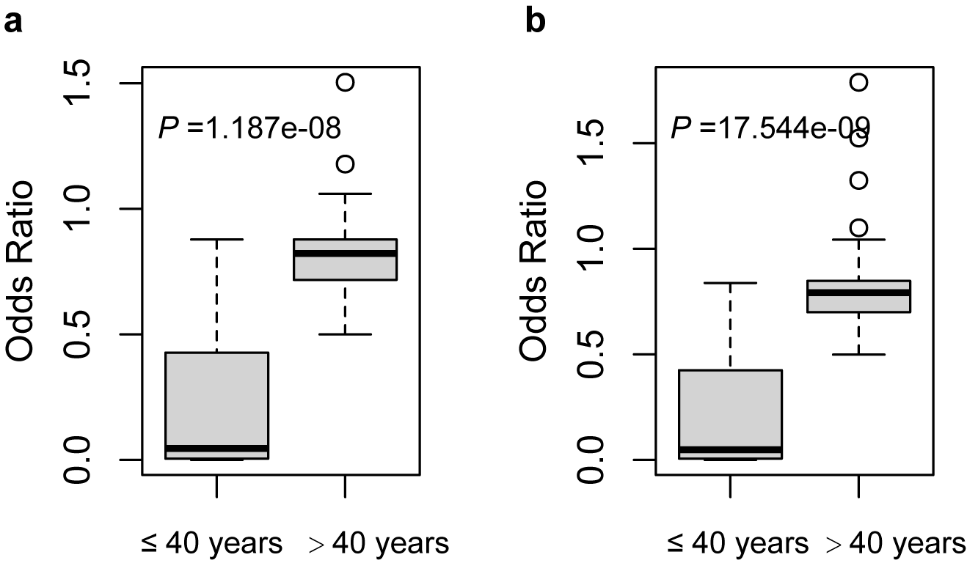


**Additional file 3.** The ORs differences before and after 40 years old in age-and sex-adjusted (a), and fully adjusted (b) models. The OR represented obesity risks of increasing per 10% abundance of *Akkermansia*. Differences between age groups were tested by Mann-Whitney *U*-test.
