# Supplementary material for: Gut bacteria Akkermansia is associated with reduced risk of obesity: evidence from the American Gut Project
Source: Nutr Metab (Lond). 2020 Oct 22;17:90. doi: 10.1186/s12986-020-00516-1 (PMC7583218; doi:10.1186/s12986-020-00516-1)
Supplement: Supplementary file 4 — Additional file 4. The effect of aging on Akkermansia-obesity associations using SWAN algorithm with 5-years-, 10-years- and 20-years-sliding windows. [file 12986_2020_516_MOESM4_ESM.docx]

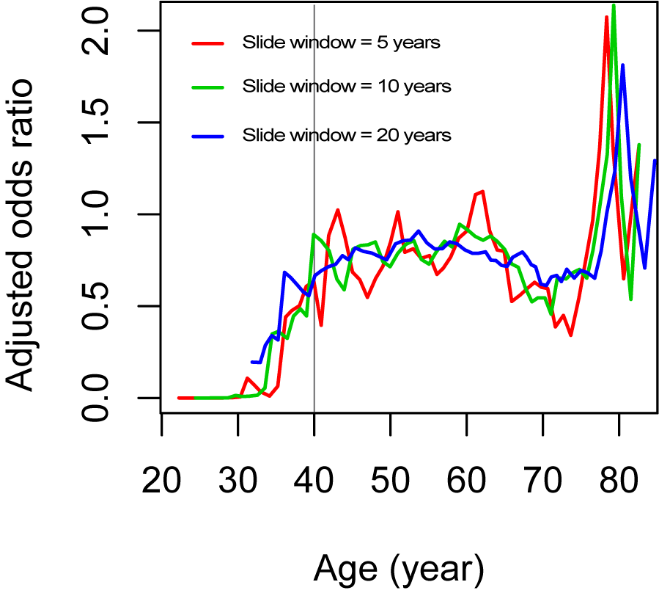


**Additional file 4.** The effect of aging on *Akkermansia-*obesity associations using SWAN algorithm with 5-years-, 10-years- and 20-years-sliding windows. The OR represented obesity risks of elevating per 10% *Akkermansia* abundance and was estimated in fully adjusted logistic regression models. Linesrepresent odds ratios and 95% CI.
